# Acute Kidney Injury and Remission of Proteinuria in Minimal Change Disease

**DOI:** 10.1016/j.ekir.2022.07.173

**Published:** 2022-08-05

**Authors:** Ryohei Yamamoto, Enyu Imai, Shoichi Maruyama, Hitoshi Yokoyama, Hitoshi Sugiyama, Asami Takeda, Shunya Uchida, Tatsuo Tsukamoto, Kazuhiko Tsuruya, Yasuhiro Akai, Kosaku Nitta, Megumu Fukunaga, Hiroki Hayashi, Tatsuya Shoji, Kosuke Masutani, Tsuneo Konta, Ritsuko Katafuchi, Saori Nishio, Takashi Wada, Shunsuke Goto, Hirofumi Tamai, Arimasa Shirasaki, Kojiro Nagai, Tomoya Nishino, Kunihiro Yamagata, Junichiro J. Kazama, Keiju Hiromura, Hideo Yasuda, Tadashi Sofue, Shouichi Fujimoto, Makoto Mizutani, Tomohiko Naruse, Takeyuki Hiramatsu, Kunio Morozumi, Hiroshi Sobajima, Yosuke Saka, Eiji Ishimura, Takafumi Ito, Daisuke Ichikawa, Takashi Shigematsu, Hiroshi Sato, Ichiei Narita, Isaka Yoshitaka, the Japan Nephrotic Syndrome Cohort Study investigators

**Affiliations:** 1 Health and Counseling Center, Osaka University, Toyonaka, Osaka, Japan; 2 Department of Nephrology, Graduate School of Medicine, Osaka University, Suita, Osaka, Japan; 3 Nakayamadera Imai Clinic, Takarazuka, Hyogo, Japan; 4 Department of Nephrology, Graduate School of Medicine, Nagoya University, Nagoya, Aichi, Japan; 5 Department of Nephrology, School of Medicine, Kanazawa Medical University, Kahoku, Ishikawa, Japan; 6 Department of Nephrology, Rheumatology, Endocrinology and Metabolism, Graduate School of Medicine Dentistry and Pharmaceutical Sciences, Okayama University, Okayama, Okayama, Japan; 7 Kidney Disease Center, Japanese Red Cross Nagoya Daini Hospital, Nagoya, Aichi, Japan; 8 Department of Internal Medicine, School of Medicine, Teikyo University, Tokyo, Japan; 9 Department of Nephrology and Dialysis, Kitano Hospital, Tazuke Kofukai Medical Research Institute, Osaka, Osaka, Japan; 10 Department of Nephrology, Nara Medical University, Kashihara, Nara, Japan; 11 First Department of Internal Medicine, Nara Medical University, Kashihara, Nara, Japan; 12 Department of Nephrology, Tokyo Women’s Medical University, Tokyo, Japan; 13 Division of Nephrology, Department of Internal Medicine, Toyonaka Municipal Hospital, Toyonaka, Osaka, Japan; 14 Department of Nephrology, School of Medicine, Fujita Health University, Toyoake, Aichi, Japan; 15 Department of Kidney Disease and Hypertension, Osaka General Medical Center, Bandaihigashi, Osaka, Osaka, Japan; 16 Division of Nephrology and Rheumatology, Department of Internal Medicine, Fukuoka University, Fukuoka, Japan; 17 Department of Cardiology, Pulmonology, and Nephrology, School of Medicine, Yamagata University, Yamagata, Japan; 18 Kidney Unit, National Hospital Organization Fukuokahigashi Medical Center, Koga, Fukuoka, Japan; 19 Division of Rheumatology, Endocrinology and Nephrology, Graduate School of Medicine, Hokkaido University, Sapporo, Hokkaido, Japan; 20 Department of Nephrology and Laboratory Medicine, Kanazawa University, Kanazawa, Ishikawa, Japan; 21 Division of Nephrology and Kidney Center, Graduate School of Medicine, Kobe University, Kobe, Hyogo, Japan; 22 Department of Nephrology, Anjo Kosei Hospital, Anjo, Aichi, Japan; 23 Department of Nephrology, Ichinomiya Municipal Hospital, Ichinomiya, Aichi, Japan; 24 Department of Nephrology, Institute of Biomedical Sciences, Tokushima University Graduate School, Kuramoto-cho, Tokushima, Japan; 25 Department of Nephrology, Nagasaki University Hospital, Nagasaki, Nagasaki, Japan; 26 Department of Nephrology, University of Tsukuba, Tsukuba, Ibaraki, Japan; 27 Department of Nephrology and Hypertension, Fukushima Medical University School of Medicine, Fukushima-City, Fukushima, Japan; 28 Department of Nephrology and Rheumatology, Gunma University Graduate School of Medicine, Maebashi, Gunma, Japan; 29 Internal Medicine, Hamamatsu University School of Medicine, Hamamatsu, Shizuoka, Japan; 30 Department of Cardiorenal and Cerebrovascular Medicine, Kagawa University, Kita-gun, Kagawa, Japan; 31 Department of Hemovascular Medicine and Artificial Organs, University of Miyazaki, Kiyotakecho, Miyazaki, Miyazaki, Japan; 32 Department of Nephrology, Handa City Hospital, Handa, Aichi, Japan; 33 Department of Nephrology, Kasugai Municipal Hospital, Kasugai, Aichi, Japan; 34 Department of Nephrology, Konan Kosei Hospital, Konan, Aichi, Japan; 35 Department of Nephrology, Masuko Memorial Hospital, Nagoya, Aichi, Japan; 36 Department of Diabetology and Nephrology, Ogaki Municipal Hospital, Ogaki, Gifu, Japan; 37 Department of Nephrology, Yokkaichi Municipal Hospital, Yokkaichi, Mie, Japan; 38 Department of Nephrology, Graduate School of Medicine, Osaka City University, Abeno-ku, Osaka, Japan; 39 Division of Nephrology, Shimane University Hospital, Izumo, Shimane, Japan; 40 Division of Nephrology and Hypertension, Department of Internal Medicine, St. Marianna University School of Medicine, Kawasaki, Kanagawa, Japan; 41 Department of Nephrology, Wakayama Medical University, Wakayama-City, Wakayama, Japan; 42 Department of Nephrology, Endocrinology, and Vascular Medicine, Gradaute School of Medicine, Tohoku University, Sendai, Miyagi, Japan; 43 Division of Clinical Nephrology and Rheumatology, Kidney Research Center, Graduate School of Medical and Dental Sciences, Niigata University, Niigata, Japan

**Keywords:** acute kidney injury, cohort study, Japan Nephrotic Syndrome Cohort Study, minimal change disease, relapse of proteinuria, remission of proteinuria

## Introduction

Minimal change disease (MCD) is one of the most common primary nephrotic syndromes. Previous studies showed that kidney survival was remarkably better in MCD than in membranous nephropathy and focal segmental glomerulosclerosis, whereas acute kidney injury (AKI) is more prevalent in MCD than in membranous nephropathy.^1^ A clinical effect of AKI in MCD remains to be elucidated. Several retrospective cohort studies reported conflicting results of an association between AKI and the incidence of remission of proteinuria in 43, 53, and 78 patients with MCD in Taiwan, Japan, and the UK, respectively. The UK study showed no association between AKI and the incidence of relapse of proteinuria.^2^, ^3^, ^4^

## Results

The Japan Nephrotic Syndrome Cohort Study is a 5-year prospective cohort study of primary nephrotic syndrome to assess the incidence of major clinical outcomes and the effectiveness of immunosuppressive therapy (IST).^5^ The present study aimed to assess the association between AKI and the incidence of remission and relapse in 113 adult patients enrolled in the Japan Nephrotic Syndrome Cohort Study aged 18 years or older, with urinary protein greater than or equal to 3.5 g/day (or urinary protein-to-creatinine ratio ≥ 3.5 g/gCr if urinary protein was missing) at IST initiation in 40 hospitals (Supplementary Figure S1). The study protocol is described in Supplementary Methods in detail.^6^ Because 108 (95.6%) and 96 (85.0%) patients achieved non-nephrotic proteinuria of urinary protein <3.5 g/day (or g/gCr) and remission within 2 months of IST, respectively, we assumed that the serum creatinine level before the onset of MCD (prepresentation serum creatinine level) was at the same serum creatinine level 2 months after initiating IST, which was used to estimate the estimated glomerular filtration rate (eGFR) before the onset of MCD (prepresentation eGFR). Based on the Kidney Disease: Improving Global Outcomes Clinical Practice Guideline for AKI,^7^ we defined the baseline AKI as an increase in baseline serum creatinine level by at least 0.3 mg/dl or 50% from prepresentation serum creatinine level. AKI stages were defined as follows: stage 1 is an increase in serum creatinine level by at least 0.3 mg/dl and/or 50% to 99%; stage 2 is an increase in serum creatinine level by 100% to 199%; and stage 3 is an increase in serum creatinine level by at least 200% and/or serum creatinine level greater than or equal to 4.0 mg/dl. We assessed the association between prepresentation AKI and the incidence of remission of proteinuria defined as urinary protein of greater than 0.3 g/day (or g/gCr) (study 1).^8^ We also assessed the clinical effect of AKI on relapse of proteinuria after remission, which was defined as urinary protein of at least 1.0 g/day (or g/ gCr) and/or dipstick urinary protein ≥2+ continued 2 or more times, in typical patients with MCD, who achieved remission within 2 months of IST (study 2).^8^

Baseline characteristics of 113 patients with MCD stratified by baseline AKI stage are listed in Table 1. The baseline AKI was observed in 37 (32.7%) patients, including 20 (17.7%), 11 (9.7%), and 6 (5.3%) patients with AKI stages 1, 2, and 3, respectively. A significant difference among 4 groups of baseline AKI stage was observed in the body mass index, systolic blood pressure, serum creatinine level, eGFR, and urinary protein level. Oral prednisolone, intravenous methylprednisolone, cyclosporin, and rituximab were administered within 1 month of IST in 111 (98.2%), 31 (27.4%), 15 (13.3%), and 2 (1.8%) patients, respectively. Prepresentation serum creatinine level and prepresentation eGFR were not significantly different among 4 groups of baseline AKI stage.

**Table 1:** Clinical characteristics of 113 adult patients with MCD stratified by AKI stage

	Baseline AKI stage at initiating IST			
Clinical variables	No-AKI	Stage 1	Stage 2	Stage 3
Number	76	20	11	6
Baseline characteristics at initiating IST				
Age, yr	45 (31, 60)	45 (30, 71)	29 (20, 43)	58 (29, 61)
Male, *n* (%)	42 (55.3)	16 (80.0)	8 (72.7)	5 (83.3)
Body mass index, kg/m^2^^a^	23.1±3.5	25.0±4.0	26.9±4.4	25.5±3.1
Systolic blood pressure, mmHg^a^	119±15	123±17	134±13	129±11
Diastolic blood pressure, mmHg	72±11	76±12	81±9	74±10
Serum albumin, g/dl	1.7±0.6	1.4±0.6	1.6±0.5	1.7±0.3
Serum creatinine, mg/dl^a^	0.74 (0.65, 0.95)	1.21 (1.08, 1.38)	2.00 (1.76, 2.91)	4.20 (3.98, 4.80)
eGFR, ml/min/1.73 m^2^^a^	80 (64, 93)	49 (41, 62)	35 (15, 44)	13 (11, 16)
≥90 mL/min/1.73 m^2^, *n* (%)	23 (30.3)	0 (0.0)	0 (0.0)	0 (0.0)
60–89	41 (53.9)	7 (35.0)	0 (0.0)	0 (0.0)
30–59	11 (14.5)	12 (60.0)	6 (54.5)	0 (0.0)
<30	1 (1.3)	1 (5.0)	5 (45.5)	6 (100.0)
Urinary protein, g/d or g/gCr^a^	7.4 (5.1, 10.3)	6.9 (5.1, 9.9)	10.5 (8.0, 15.6)	9.5 (8.0, 11.7)
RAS blockade, *n* (%)	9 (11.8)	5 (25.0)	2 (18.2)	0 (0.0)
Estimated kidney function before MCD presentation (= kidney function 2 mo after IST initiation)				
Prepresentation serum creatinine, mg/dl	0.81±0.30	0.83±0.20	0.98±0.34	0.98±0.24
Prepresentation eGFR, ml/min/1.73 m^2^	81±23	83±28	78±33	66±17
≥90 mL/min/1.73 m^2^, *n* (%)	25 (32.9)	7 (35.0)	4 (36.4)	0 (0.0)
60–89	37 (48.7)	9 (45.0)	4 (36.4)	3 (50.0)
30–59	13 (17.1)	4 (20.0)	2 (18.2)	3 (50.0)
<30	1 (1.3)	0 (0.0)	1 (9.1)	0 (0.0)
Immunosuppressive drugs within 1 mo of IST				
Oral prednisolone, *n* (%)	74 (97.4)	20 (100.0)	11 (100.0)	6 (100.0)
Intravenous methylprednisolone, *n* (%)	17 (22.4)	5 (25.0)	6 (54.5)	3 (50.0)
Cyclosporin, *n* (%)	9 (11.8)	4 (20.0)	2 (18.2)	0 (0.0)
Rituximab, *n* (%)	2 (2.6)	0 (0.0)	0 (0.0)	0 (0.0)
Dialysis before and within 2 mo of IST				
Dialysis before IST, *n* (%)	0 (0.0)	0 (0.0)	0 (0.0)	1 (16.7)
Dialysis within 2 mo of IST, *n* (%)	1 (1.3)	2 (10.0)	2 (18.2)	1 (16.7)
Outcomes after initiating IST and total observational period				
Total observational period, yr	4.9 (3.3, 5.0)	4.9 (2.9, 5.0)	5.0 (3.2, 5.0)	5.0 (4.9, 5.0)
Remission, *n* (%)	72 (94.7)	20 (100.0)	11 (100.0)	6 (100.0)
Remission within 2 mo of IST, *n* (%)	68 (89.5)	17 (85.0)	7 (63.6)	4 (66.7)
Relapse after remission, *n* (%)^b^	29 (42.6)	8 (47.1)	3 (42.9)	2 (50.0)
End stage kidney disease, *n* (%)	2 (2.6)^c^	0 (0.0)	0 (0.0)	0 (0.0)

AKI, acute kidney injury; eGFR, estimated glomerular filtration rate; IST, immunosuppressive therapy; MCD, minimal change disease; RAS, renin-angiotensin system.

Mean±SD; median (25%, 75%).

a *P* < 0.05.

b Including 96 patients with remission within 2 months of IST.

c Including a patient with remission 8 days after initiating IST and relapse 1.5 years after remission, who started kidney replacement therapy 2.0 years after initiating IST, and another patient without remission, who started kidney replacement therapy 1.2 years after initiating IST.

In study 1, during the median observational period of 15 days (interquartile range 10–32), the incidence of remission of proteinuria, was observed in 72 (94.7%), 20 (100.0%), 11 (100.0%), 6 (100.0%) patients with no-AKI, AKI stages 1, 2, and 3 groups, respectively (Table 1). In the no-AKI group, 2 patients progressed to end stage kidney disease 1.2 and 2.0 years after initiating IST. Patients with a higher baseline AKI stage were more likely to have a lower cumulative probability of remission (*P*_trend_ = 0.010, Supplementary Figure S2A), whereas prepresentation eGFR was not significantly associated with the incidence of remission (*P*_trend_ = 0.058, Supplementary Figure S2B). An unadjusted model showed that patients with baseline AKI stage 2 had a significantly lower cumulative probability of remission than those without AKI (Table 2). After the multivariable adjustment, baseline AKI stages 2 and 3 were identified as predictors of late remission (adjusted hazard ratio [95% confidence interval] of no-AKI and AKI stages 1, 2, and 3:1.00 [reference], 0.80 [0.47, 1.36], 0.33 [0.16, 0.70], and 0.39 [0.15, 0.97], respectively), along with age and serum albumin level, whereas prepresentation eGFR was not a predictor of late remission.

**Table 2:** Predictors of remission and relapse after remission

	Remission (*n* = 113)		Relapse after remission (*n* = 96)	
Predictors	Unadjusted HR (95% CI)	Adjusted HR (95% CI)	Unadjusted HR (95% CI)	Adjusted HR (95% CI)
Age, per 10 yr	0.87 (0.78, 0.96)^a^	0.82 (0.71, 0.94)^a^	0.92 (0.77, 1.09)	0.92 (0.72, 1.16)
Male	1.15 (0.78, 1.72)	1.35 (0.88, 2.09)	0.80 (0.44, 1.48)	0.58 (0.27, 1.25)
Body mass index, per 1.0 kg/m^2^	0.96 (0.91, 1.00)	0.95 (0.90, 1.01)	1.03 (0.95, 1.12)	1.02 (0.93, 1.13)
Systolic blood pressure, per 10 mmHg	0.89 (0.80, 0.99)^a^	0.97 (0.84, 1.13)	0.98 (0.78, 1.22)	1.05 (0.80, 1.39)
Serum albumin, per 1.0 g/dl	0.69 (0.48, 1.00)	0.63 (0.42, 0.95)^a^	0.80 (0.47, 1.34)	0.78 (0.42, 1.43)
UP, per 1.0 Log g/d or g/gCr	1.07 (0.76, 1.53)	1.40 (0.93, 2.11)	1.23 (0.68, 2.22)	0.97 (0.48, 1.95)
Prepresentation eGFR				
>90 ml/min/1.73 m^2^	1.00 (reference)	1.00 (reference)	1.00 (reference)	1.00 (reference)
60–89	1.10 (0.72, 1.72)	1.55 (0.93, 2.60)	1.19 (0.60, 2.36)	1.61 (0.59, 3.77)
<60	0.59 (0.35, 1.01)	1.44 (0.67, 3.08)	0.69 (0.26, 1.82)	0.84 (0.21, 3.44)
AKI stage				
No-AKI	1.00 (reference)	1.00 (reference)	1.00 (reference)	1.00 (reference)
Stage 1	0.84 (0.51, 1.38)	0.80 (0.47, 1.36)	1.28 (0.59, 2.80)	1.36 (0.56, 3.33)
Stage 2	0.52 (0.27, 0.98)^a^	0.33 (0.16, 0.70)^a^	1.24 (0.38, 4.08)	1.18 (0.33, 4.25)
Stage 3	0.48 (0.21, 1.12)	0.39 (0.15, 0.97)^a^	1.04 (0.25, 4.35)	0.93 (0.20, 4.34)
Intravenous mPSL within 1 mo of IST	0.66 (0.43, 1.01)	0.71 (0.45, 1.13)	1.04 (0.52, 2.07)	1.12 (0.52, 2.41)
Cyclosporine within 1 mo of IST	0.56 (0.32, 0.99)^a^	0.64 (0.34, 1.20)	0.93 (0.37, 2.38)	1.27 (0.46, 3.52)

AKI, acute kidney injury; CI, confidence interval; eGFR, estimated glomerular filtration rate; HR, hazard ratio; IST, immunosuppressive therapy; mPSL, methyprednisolone; UP, urinary protein.

a *P* < 0.05.

In study 2, among 96 patients who achieved remission within 2 months of IST, including 68, 17, 7, and 4 patients of no-AKI, AKI stages 1, 2, and 3 groups, respectively, the incidence of relapse of proteinuria was observed in 42 (43.8%) patients during the median observational period of 2.3 years (interquartile range 0.9, 4.6) after remission. AKI stage and prepresentation eGFR were not associated with the incidence of relapse in the unadjusted and adjusted models (Table 2).

## Discussion

Several small retrospective cohort studies have assessed the association between AKI and remission of proteinuria among adult patients with MCD. A retrospective cohort study, including 53 adult patients with MCD in Taiwan, reported that 25 patients with AKI defined as at least a 35% decline in creatinine clearance at kidney biopsy, had a significantly lower cumulative incidence of remission than 28 patients without AKI.^2^ In contrast, another retrospective cohort study, including 78 patients with MCD in the UK, showed that AKI at kidney biopsy defined as at least a 50% increase in serum creatinine level was not associated with the cumulative incidence of remission.^4^ Nevertheless, these studies did not control for the potential confounding factors, including age.^9^ A Japanese retrospective cohort study assessed the association between AKI and the incidence of remission, after adjusting for clinically relevant factors.^3^ This study elaborately defined AKI as an increase in serum creatinine level by at least 0.3 mg/dl within 48 hours and an increase in serum creatinine level to at least 1.5 times the baseline level known or presumed to have occurred within the prior 7 days during the 4 weeks after initiating IST. After categorizing patients into 3 groups, namely, no-AKI, AKI stage 1 or 2, and AKI stage 3, according to the Kidney Disease: Improving Global Outcomes classification, this study clarified a dose-dependent association between AKI stage and the incidence of remission in multivariable-adjusted Cox proportional hazards models. Even patients with AKI stage 1 or 2 had significantly lower cumulative probability of remission, compared to those without AKI. In addition, the larger sample size of the present study enabled us to identify AKI stage 2, not stage 1, as a significant suppressor of remission (Table 2).

One of the potential biases in the present study was that patients with baseline AKI and no improvement in eGFR 2 months after initiating IST were incorrectly categorized into the no-AKI group. Given that AKI delayed remission of MCD, this misclassification suppressed the remission in the no-AKI group and promoted the remission in the AKI group, thereby underestimating the clinical effect of AKI on remission of proteinuria. The true effect of AKI might be stronger than that observed in the present study.

In conclusion, the Japan Nephrotic Syndrome Cohort Study identified AKI stage 2 or higher as a significant suppressor of remission in adult patients with MCD. Nevertheless, because of the nature of the observational study design of the Japan Nephrotic Syndrome Cohort Study, the clinical effect of AKI in MCD should be confirmed in large well-designed studies.

## Disclosure

All the authors declared no competing interests.

## Appendix

### List of the Japan Nephrotic Syndrome Cohort Study (JNSCS)

JNSCS has been supported by a large number of investigators in 55 participating facilities as follows: Hokkaido University Hospital, Sapporo, Hokkaido (Saori Nishio, Yasunobu Ishikawa, Daigo Nakazawa, and Tasuku Nakagaki); JCHO Sendai Hospital, Sendai, Miyagi (Toshinobu Sato, Mitsuhiro Sato, and Satoru Sanada); Tohoku University Hospital, Sendai, Miyagai (Hiroshi Sato, Mariko Miyazaki, Takashi Nakamichi, Tae Yamamoto, Kaori Narumi, and Gen Yamada); Yamagata University Hospital, Yamagata, Yamagata (Tsuneo Konta, and Kazuobu Ichikawa); Fukushima Medical University Hospital, Fukushima, Fukushima (Junichiro James Kazama, Tsuyoshi Watanabe, Koichi Asahi, Yuki Kusano, and Kimio Watanabe); University of Tsukuba Hospital, Tsukuba, Ibaraki (Kunihiro Yamagata, Joichi Usui, Shuzo Kaneko, and Tetsuya Kawamura); Gunma University Hospital, Maeashi, Gunma (Keiju Hiromura, Akito Maeshima, Yoriaki Kaneko, Hidekazu Ikeuchi, Toru Sakairi, and Masao Nakasatomi); Saitama Medical Center, Saitama Medical University, Kawagoe, Saitama (Hajime Hasegawa, Takatsugu Iwashita, Taisuke Shimizu, Koichi Kanozawa, Tomonari Ogawa, Kaori Takayanagi, and Tetsuya Mitarai); Saitama Medical University, Irumagun, Saitama (Hirokazu Okada, Tsutomu Inoue, Hiromichi Suzuki, and Kouji Tomori); Tokyo Women’s Medical University, Shinjuku-ku, Tokyo (Kosaku Nitta, Takahito Moriyama, Akemi Ino, and Masayo Sato); Teikyo University School of Medicine, Itabashi-ku, Tokyo (Shunya Uchida, Hideaki Nakajima, Hitoshi Homma, Nichito Nagura, Yoshifuru Tamura, Shigeru Shibata, and Yoshihide Fujigaki); Juntendo Faculty of Medicine, Bunkyo-ku, Tokyo (Yusuke Suzuki, Yukihiko Takeda, Isao Osawa, and Teruo Hidaka); St. Marianna University, Kawasaki, Kanagawa (Yugo Shibagaki, Sayuri Shirai, Daisuke Ichikawa, Tsutomu Sakurada, Tomo Suzuki, and Mikako Hisamichi); Niigata University Medical and Dental Hospital, Niigata, Niigata (Ichiei Narita, Naohumi Imai, Yumi Ito, Shin Goto, Yoshikatsu Kaneko, and Rhohei Kaseda); Kanazawa Medical University, Uchinada, Ishikawa (Hitoshi Yokoyama, Keiji Fujimoto, and Norifumi Hayashi); Kanazawa Univeristy Hospital, Kanazawa, Ishikawa (Takashi Wada, Miho Shimizu, Kengo Furuichi, Norihiko Sakai, Yasunori Iwata, Tadashi Toyama, and Shinji Kitajima); National Hospital Organization Kanazawa Medical Center, Kanazawa, Ishikawa (Kiyoki Kitagawa); Ogaki Municipal Hospital, Ogaki, Gifu (Hiroshi Sobajima, Norimi Ohashi, So Oshitani, and Kiyohito Kawashima); Gifu Prefectural Tajimi Hospital, Tajimi, Gifu (Tetsushi Mimura); Hamamatsu University Hospital, Hamamatsu, Shizuoka (Hidemo Yasuda, Akira Hishida, and Yoshihide Fujigaki); Shizuoka General Hospital, Shizuoka, Shizuoka (Satoshi Tanaka, and Noriko Mori); Chutoen General Medical Center, Kakegawa, Shizuoka (Toshiyuki Akahori, and Yutaka Fujita); Nagoya University Graduate School of Medicine, Nagoya, Aichi (Shoichi Maruyama, Naotake Tsuboi, Tomoki Kosugi, Takuji Ishimoto, Takayuki Katsuno, Noritoshi Kato, and Waichi Sato); Japanese Red Cross Nagoya Daini Hospital, Nagoya, Aichi (Asami Takeda, Kunio Morozumi, Yasuhiro Ohtsuka, Hibiki Shinjo, and Akihito Tanaka); Fujita Health University School of Medicine, Toyoake, Aichi (Hiroki Hayashi, Yukio Yuzawa, Midori Hasegawa, Daijo Inaguma, Shigehisa Koide, and Kazuo Takahashi); Konan Kosei Hospital, Konan, Aichi (Takeyuki Hiramatsu, Shinji Furuta, and Hideaki Ishikawa); Anjo Kosei hospital, Anjo, Aichi (Hirofumi Tamai, and Takatoshi Morinaga); Ichinomiya Municipal Hospital, Ichinomiya, Aichi (Arimasa Shirasaki, Toshiki Kimura, and Mina Kato); Japanese Red Cross Nagoya Daiichi Hospital, Nagaoya, Aichi (Shizunori Ichida, and Nobuhide Endo); Kasugai Municipal Hospital, Kasugai, Aichi (Tomohiko Naruse, Yuzo Watanabe, and Yosuke Saka); Kainan hospital, Yatomi, Aichi (Satashi Suzuki, Michiko Yamazaki, and Rieko Morita); Masuko Memorial Hospital, Nagoya, Aichi (Kunio Morozumi, Kunio Morozumi, Kaoru Yasuda, Chika Kondo, Takahiro Morohiro, Rho Sato, and Yuichi Shirasawa); Chubu Rosai Hospital, Nagoya, Aichi (Yoshiro Fujita, Hideaki Shimizu, and Tatsuhito Tomino); Handa City Hospital, Handa, Aichi (Makoto Mizutani); Yokkaichi Municipal Hospital, Yokkaichi, Mie (Yosuke Saka, Hiroshi Nagaya, and Makoto Yamaguchi); Kitano Hospital, Osaka, Osaka (Tatsuo Tsukamoto, Eri Muso, Hiroyuki Suzuki, Tomomi Endo, and Hiroko Kakita); Toyonaka Municipal Hospital, Toyonaka, Osaka (Megumu Fukunaga); Osaka General Medical Center, Osaka, Osaka (Tatsuya Shoji, and Terumasa Hayashi); Osaka City University Hospital, Osaka, Osaka (Eiji Ishimura, Akihiro Tsuda, Shinya Nakatani, Ikue Kobayashi, Mitsuru Ichii, Akinobu Ochi, and Yoshiteru Ohno); Osaka Univeristy hospital, Suita, Osaka (Yoshitaka Isaka, Enyu Imai, Yasuyuki Nagasawa, Hirotsugu Iwatani, Ryohei Yamamoto, and Tomoko Namba); Kobe University hospital, Kobe, Hyogo (Shunsuke Goto MD, and Shinichi Nishi); Nara Medical University Hospital, Kashihara, Nara (Yasuhiro Akai, Ken-ichi Samejima, Masaru Matsui, Miho Tagawa, Kaori Tanabe, and Hideo Tsushima); Wakayama Medical University Hospital, Wakayama, Wakayama (Takashi Shigematsu, Masaki Ohya, Shigeo Negi, and Toru Mima); Shimane University Hospital, Izumo, Shimane (Takafumi Ito); Okayama Univeristy Hospital, Okayama, Okayama (Hitoshi Sugiyama, Keiko Tanaka, Toshio Yamanari, Masashi Kitagawa, Akifumi Onishi, and Koki Mise); Kawasaki Medical School, Kurashiki, Okayama (Naoki Kashihara, Tamaki Sasaki, Sohachi Fujimoto, and Hajime Nagasu); Graduate School of Medicine, The University of Tokushima,Tokushima,Tokushima (Kojiro Nagai, and Toshio Doi); Kagawa University, Miki-cho, Takamatsu, Japan (Tadashi Sofue, Hideyasu Kiyomoto, Kumiko Moriwaki, Taiga Hara, Yoko Nishijima, Yoshio Kushida, and Tetsuo Minamino); Kochi Medical School, Kochi University, Nankoku, Kochi (Yoshio Terada, Taro Horino, Yoshinori Taniguchi, Kosuke Inoue, Yoshiko Shimamura, and Tatsuki Matsumoto); Kyushu University Hospital, Fukuoka, Fukuoka (Kazuhiko Tsuruya, Hisako Yoshida, Naoki Haruyama, Shunsuke Yamada, Akihiro Tsuchimoto, and Yuta Matsukuma); Fukuoka University Hospital, Fukuoka, Fukuoka (Kosuke Masutani, Yasuhiro Abe, Aki Hamauchi, Tetsuhiko Yasuno, and Kenji Ito); Kurume University Hospital, Kurume, Fukuoka (Kei Fukami, Junko Yano, Chika Yoshida, Yuka Kurokawa, and Nao Nakamura); National Fukuoka Higashi Medical Center, Koga, Fukuoka (Ritsuko Katafuchi, Hiroshi Nagae, Shumei Matsueda, and Kazuto Abe); Nagasaki University Hospital, Nagasaki, Nagasaki (Tomoya Nishino, Tadashi Uramatsu, and Yoko Obata); and Miyazaki University Hospital, Miyazaki, Miyazaki (Shouichi Fujimoto, Yuji Sato, Masao Kikuchi, Ryuzo Nishizono, Takashi Iwakiri, and Hiroyuki Komatsu).

## Supplementary Material

**Supplementary Methods.**

**Figure S1.** Flow diagram of the inclusion and exclusion of study participants

**Figure S2.** Cumulative probability of the incidence of complete remission stratified by (A) AKI stage and (B) prepresentation eGFR category
