# Supplementary material for: Acute Kidney Injury and Remission of Proteinuria in Minimal Change Disease
Source: Kidney Int Rep. 2022 Aug 5;7(10):2283–8. doi: 10.1016/j.ekir.2022.07.173 (PMC9546736; doi:10.1016/j.ekir.2022.07.173)
Supplement: Supplementary File (PDF) [file mmc1.pdf]

Supplementary Material

Supplementary methods

Figure S1. Flow diagram of the inclusion and exclusion of study participants.

Figure S2. Cumulative probability of the incidence of complete remission stratified by AKI stage (a) and pre-presentation eGFR category (b).

Supplementary methods

Participants

The JNSCS is a 5-year prospective cohort study of primary nephrotic syndrome to assess the incidence of major clinical outcomes and the effectiveness of immunosuppressive therapy (IST). The details of the study design have been described elsewhere.[1,2] Briefly, 374 patients diagnosed as primary nephrotic syndrome using kidney biopsy between 2009 and 2010 in 55 hospitals in Japan were enrolled in the JNSCS. Of 155 patients with MCD, 113 adult patients aged ≥18 years with urinary protein of ≥3.5 g/day (or urinary protein-to-creatinine ratio [UPCR] of ≥3.5 g/gCr if urinary protein was missing) at IST initiation in 40 hospitals were included in the present study, after excluding 2 patients without IST during the observational period, 16 patients aged <18 years, 17 patients with urinary protein of <3.5 g/day (or g/gCr) at IST initiation, and 3 patients with missing baseline data at IST initiation, 2 patients lost to follow-up within 2 months of IST, and 2 patients with missing data of serum creatinine 2 months after IST initiation (Figure 1). To assess the clinical impact of AKI on relapse of proteinuria, 96 patients with remission within 2 months of IST, which was defined as urinary protein of <0.3 g/day (or g/gCr),[3] were included after excluding 17 patients without remission within 2 months of IST.

The ethics committee of Osaka University Hospital (approval number 17035-4) and the institutional review board of each participating hospital approved the study protocol for the JNSCS. All procedures performed in the JNSCS involving human participants were in accordance with the ethical standards of the institutional research committee at which the studies were conducted and with the 1964 Declaration of Helsinki and its later amendments or comparable ethical standards. Informed consent was obtained from participants in 53 hospitals and a single hospital used an opt-out approach to provide informed consent, according to Japanese Ethical Guidelines for Medical and Health Research Involving Human Subjects.

Measurements

The baseline characteristics at IST initiation included age; sex; body mass index; systolic and diastolic blood pressure; serum creatinine and albumin concentrations; eGFR; urinary protein; and renin-angiotensin system (RAS) blockade. To calculate eGFR, the Japanese equation was used: $\text{eGFR} = 194 \times \text{age (year)}^{-0.287} \times \text{serum creatinine (mg/dL)}^{-0.094} \times 0.739$ (if female).[4] We also collected the use of immunosuppressive drugs during the 1-month period after initiating IST, including oral prednisolone, intravenous methylprednisolone (mPSL), cyclosporin, tacrolimus, cyclophosphamide, mycophenolate mofetil, mizoribine, and rituximab.

Because 108 (95.6%) and 96 (85.0%) patients achieved non-nephrotic proteinuria of UP <3.5 g/day (or g/gCr) and remission within 2 months of IST, respectively, we assumed that the serum creatinine level before the onset of MCD (pre-presentation serum creatinine level) was at the same serum creatinine level 2 months after initiating IST, which was used to estimate the eGFR before the onset of MCD (pre-presentation eGFR). Based on the KDIGO Clinical Practice Guideline for AKI,[5] we defined the baseline AKI as an increase in baseline serum creatinine level by ≥0.3 mg/dL or 50% from pre-presentation serum creatinine level. AKI stages were defined as follows: stage 1, an increase in serum creatinine level by ≥0.3 mg/dL and/or 50%–99%; stage 2, an increase in serum creatinine level by 100%–199%; and stage 3, an increase in serum creatinine level by ≥200% and/or serum creatinine level ≥4.0 mg/dL.

The outcomes were (i) remission of proteinuria, as defined above, and (ii) relapse of proteinuria defined as urinary protein ≥1.0 g/day (or g/ gCr) and/or dipstick urinary protein ≥2+

continued two or more times.[3] To assess the association between baseline AKI and the incidence of remission, the observational period was defined as the period from the IST initiation to the incidence of remission, or the last UP measurement, whichever came first. To assess the association between baseline AKI and the incidence of relapse, the observational period was defined as the period from the incidence of remission to the incidence of relapse or the last urinary protein measurement, whichever came first. We also collected the incidence of ESKD requiring kidney replacement therapy during the observational period. The total observational period was defined as the period from IST initiation to the incidence of ESKD or the last measurement of serum creatinine, whichever came first.

Statistics

The clinical characteristics of patients among the baseline AKI stages were compared using analysis of variance, the Kruskal-Wallis test, and the Fisher's exact test, as appropriate. Pearson's rho was calculated to assess the correlation of baseline AKI stage with baseline eGFR and pre-presentation eGFR.

Cumulative probabilities of the incidence of remission in the four groups of baseline AKI stage were calculated using the Kaplan-Meire method and compared using the log-rank test and the log-rank test for trend. To assess the associations between baseline AKI stage and the incidence of remission and relapse, we used unadjusted and multivariable-adjusted Cox proportional hazards models. Covariates included age (years), sex, body mass index (kg/m^2), systolic blood pressure (mmHg), serum albumin concentration (g/dL), urinary protein level (Log g/day or g/gCr), and AKI stage (no AKI, AKI stage 1, 2, and 3) at the IST initiation; pre-presentation eGFR (<60, 60–89, and ≥90 mL/min/1.73 m^2); and use of intravenous mPSL and cyclosporin within 1 month of IST. Urinary protein level was logarithmically transformed because of its skewed distribution. Owing to the small number of patients with pre-presentation eGFR <30 mL/min/1.73 m^2 (n=2), the patients with pre-presentation eGFR <30 and 30–59 mL/min/1.73 m^2 were classified into a single category.

Continuous variables were expressed as the mean ± standard deviation or median and interquartile range (IQR), as appropriate, and categorical variables were expressed as a numbers and proportions. A *P*-value <0.05 was considered statistically significant. Statistical analyses were performed using Stata, version 17.0 (Stata Corp, www.stata.com) and R, version 4.1.2 (The R Foundation for Statistical Computing, https://www.r-project.org/).

References

1. Yamamoto R, Imai E, Maruyama S, et al. Regional variations in immunosuppressive therapy in patients with primary nephrotic syndrome: the Japan nephrotic syndrome cohort study. Clin Exp Nephrol. 2018;22(6):1266-1280.
2. Yamamoto R, Imai E, Maruyama S, et al. Incidence of remission and relapse of proteinuria, end-stage kidney disease, mortality, and major outcomes in primary nephrotic syndrome: the Japan Nephrotic Syndrome Cohort Study (JNSCS). Clin Exp Nephrol. 2020;24(6):526-540.
3. Nishi S, Ubara Y, Utsunomiya Y, et al. Evidence-based clinical practice guidelines for nephrotic syndrome 2014. Clin Exp Nephrol. 2016;20(3):342-370.
4. Matsuo S, Imai E, Horio M, et al. Revised Equations for Estimated GFR From Serum Creatinine in Japan. Am J Kidney Dis. 2009;53(6):982-992.
5. Doi K, Nishida O, Shigematsu T, et al. The Japanese Clinical Practice Guideline for acute kidney injury 2016. J Intensive Care Med. 2018;6(1):1-55.

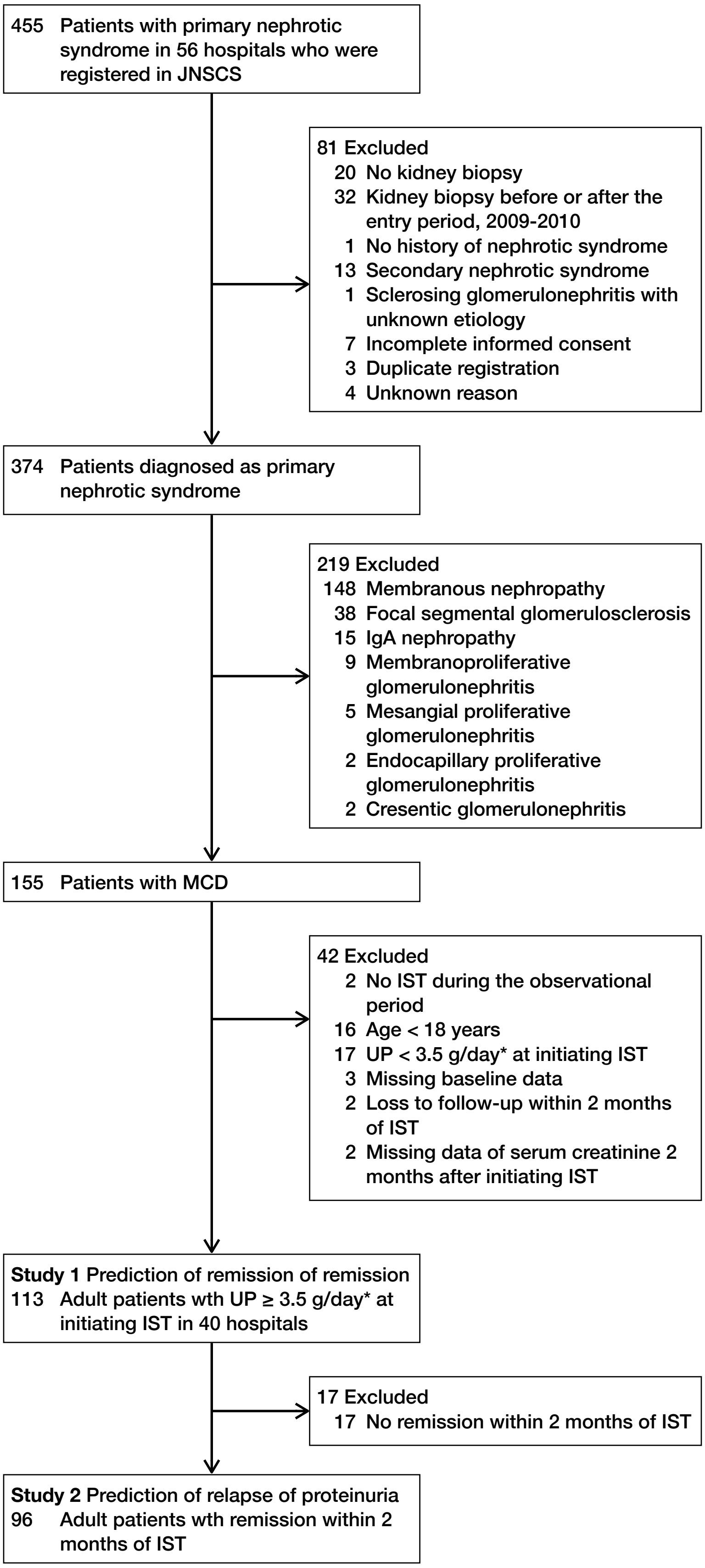

Figure S1. Flow diagram of the inclusion and exclusion of study participants.

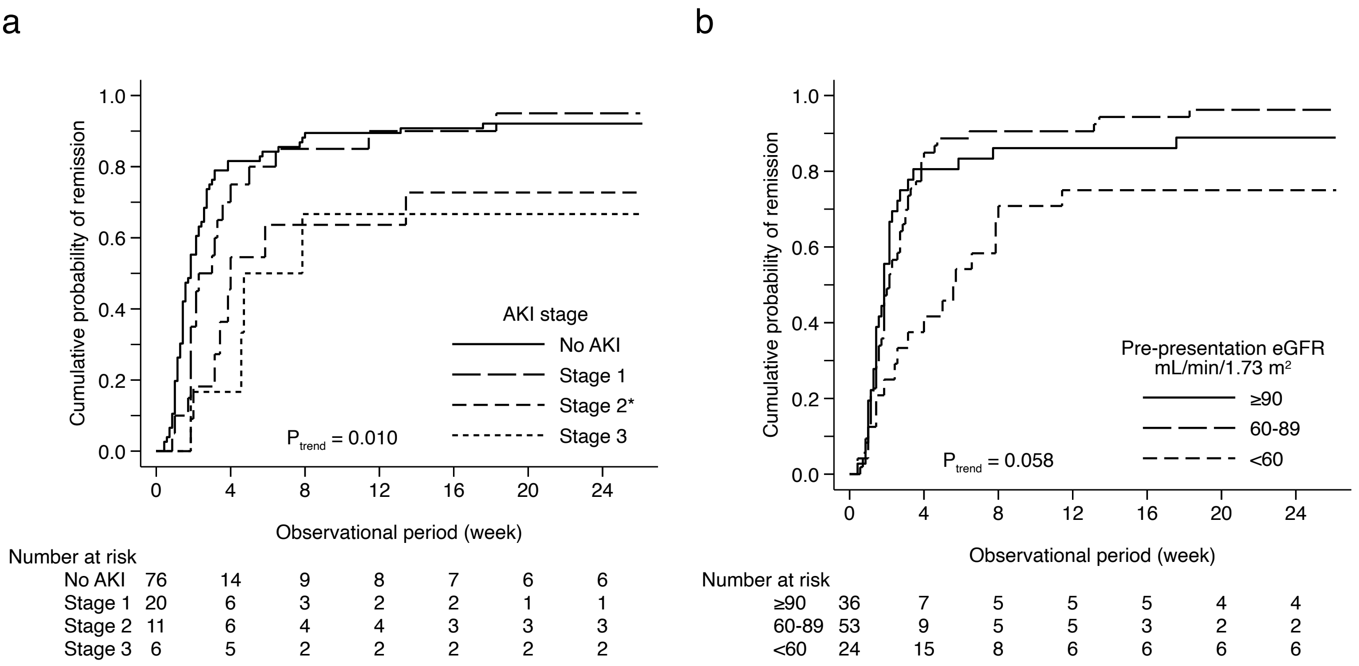

Figure S2. Cumulative probability of the incidence of complete remission stratified by AKI stage (a) and pre-presentation eGFR category (b).

AKI, acute kidney injury; eGFR, estimated glomerular filtration rate

*P <0.05 vs. no AKI (a) and pre-presentation eGFR of ≥90 mL/min/1.73 m^2 (b)
